# Gray Matter Volume in the Developing Frontal Lobe and Its Relationship With Executive Function in Late Childhood and Adolescence: A Community-Based Study

**DOI:** 10.3389/fpsyt.2021.686174

**Published:** 2021-07-13

**Authors:** Dajung Sung, Bumhee Park, Bora Kim, Hayeon Kim, Kyu-In Jung, Seung-Yup Lee, Bung-Nyun Kim, Subin Park, Min-Hyeon Park

**Affiliations:** ^1^Department of Psychiatry, College of Medicine, Eunpyeong St. Mary's Hospital, The Catholic University of Korea, Seoul, South Korea; ^2^Department of Biomedical Informatics, Ajou University School of Medicine, Suwon, South Korea; ^3^Office of Biostatistics, Ajou Research Institute for Innovative Medicine, Ajou University Medical Center, Suwon, South Korea; ^4^Department of Psychiatry and Behavioral Science, Seoul National University College of Medicine, Seoul, South Korea; ^5^Department of Research Planning, National Center for Mental Health, Seoul, South Korea

**Keywords:** frontal lobe, executive functions, late childhood, adolescence, attentional networks

## Abstract

**Background:** During late childhood and adolescence, the frontal lobe undergoes critical developmental changes, affecting a wide range of executive functions significantly. Conversely, abnormality in the maturation of the frontal lobe during this period may result in a limited ability to effectively use various executive functions. However, at present, it is still unclear how the structural development of the frontal lobe is associated with different aspects of executive functions during this developmental period. To fill the gap in evidence, we aimed to elucidate gray matter volume (GMV) in the frontal lobe and its relationship with multiple aspects of executive functions in late childhood and adolescence.

**Methods:** We recruited our participants aged between 6 and 17 years to assess GMV in the frontal lobe and its relationship with different domains of executive functions in late childhood and adolescence. We used the voxel-based morphometry–DARTEL procedure to measure GMVs in multiple frontal sub-regions and Stroop test and Advanced Test of Attention (ATA) to measure executive functions. We then conducted partial correlation analyses and performed multiple comparisons with different age and sex groups.

**Results:** Overall, 123 participants took part in our study. We found that many regional GMVs in the frontal lobe were negatively correlated with ATA scores in participants in late childhood and positively correlated with ATA scores in participants in adolescence. Only a few correlations of the GMVs with Stroop test scores were significant in both age groups. Although most of our results did not survive false discovery rate (FDR) correction (i.e., FDR <0.2), considering their novelty, we discussed our results based on uncorrected *p*-values. Our findings indicate that the frontal sub-regions that were involved in attentional networks may significantly improve during late childhood and become stabilized later in adolescence. Moreover, our findings with the Stroop test may also present the possibility of the later maturation of higher-order executive functioning skills.

**Conclusion:** Although our findings were based on uncorrected *p*-values, the novelty of our findings may provide better insights into elucidating the maturation of the frontal lobe and its relationship with the development of attention networks in late childhood and adolescence.

## Introduction

The development of the frontal lobe has drawn much attention to neuroimaging and developmental studies because of its strong association with a wide range of executive functions (EFs), from basic motor movements to complex decision making (1). The frontal lobe is known to manage various EFs that are essential for performing complex tasks including the selection and perception of information, inhibitory control, maintenance, working memory, and self-directed behavior (2). A large body of longitudinal neuroimaging studies have exhibited that the frontal lobe is among the last regions of the brain to mature, and that it may not fully maturate until halfway through the third decade of life (3, 4). Especially during childhood and adolescence, the frontal lobe undergoes profound neurodevelopmental changes due to the pruning of excess synapses (5). According to a well-established body of magnetic resonance imaging (MRI) studies (4, 6–9), there is a decrease in gray matter density with a non-linear pattern in the frontal cortices throughout adolescence (9). A longitudinal study with a wide age range (between 4 and 22 years of age) has demonstrated that gray matter volume (GMV) in the frontal lobe increases during preadolescence, especially in the pre-frontal cortex (8, 10). Within the anatomical structure of the frontal lobe, GMV in the precentral gyrus develops earlier than other frontal sub-regions, whereas the superior and inferior frontal gyri maturate later (9). Furthermore, there has been robust evidence on sex differences in total intracranial volume (TIV) across different ages (11, 12). For example, in the developing brain, GMVs in many regions in the frontal lobe reach maximum volume at around 12 years for males and 11 years for females (10, 13). Following late childhood, the GMV in the frontal lobe gradually decreases in both males, and females (13).

Such structural maturation of the frontal lobe also leads to significant development of EFs throughout childhood and adolescence (14, 15). An extensive body of neuroimaging studies have demonstrated that the main domains (i.e., shifting, working memory, inhibition) of EFs emerge during the first few years of life, and their refinement and stabilization continue throughout adolescence (15, 16). That is, some cognitive abilities may develop early, but a range of higher-order executive functioning skills does not reach their peak until post-adolescence (17–19). Higher-order executive functioning skills are defined as multidimensional executive and control processes such as reasoning, planning, organizing, problem-solving, sustained attention, response inhibition, and cognitive flexibility (20–22). Anderson et al. (23) examined how children and adolescents aged between 11 and 17 years performed differently on a variety of EF tasks. They observed that there was an improvement in some EF task performances that required selective attention, working memory, and problem-solving in the group of adolescents. They also found that attentional control-processing speed had the most significant development in this age group (23). They speculated that their findings might be linked to the neurobiological processes of pruning and myelination in the frontal lobe, particularly during this developmental period. Moreover, this might reflect that the main domains of EFs have their own developmental trajectories. Similarly, an earlier cognitive study examined EF skills including working memory and inhibition in three age groups (i.e., 6–7, 8–10, and 11–12 years) and found significant differences in the performances of the age groups in EF tasks (24). Explicitly, there was a remarkable peak in the tasks that assessed planning and processing speed in the group of 11 to 12-year-old children (24).

Although there has been a substantial increase in understanding of this area across different ages over the past two decades, yet little is known is about how the neurocognitive development of the frontal lobe is related to the different domains of EFs in children, and adolescents in the general population. Abnormality in the maturation of the frontal lobe in late childhood and adolescence may result in a limited ability to effectively use the domains of EFs (23). This is of particular importance because the transition from late childhood to adolescence is regarded as a time where some externalizing problems [e.g., attention-deficit/hyperactivity disorder (ADHD)] can possibly transform into more severe behavioral forms including oppositional defiant disorder, vandalism, theft, physical aggression, delinquency, and bullying (25–27). Furthermore, this developmental period is associated with increased risk-taking behaviors (e.g., substance use) and various mental health problems (e.g., depression, schizophrenia, bipolar disorders) (15, 28). Recently, Straub et al. (29) examined GMVs in a relatively large sample of depressed and healthy youth and found greater GMVs in the dorsolateral pre-frontal cortex in the group of depressed youth. Replicating findings from previous studies (30–32), they suggested that depressed youth may be more vulnerable to delayed brain maturation.

Although abnormality in the neurocognitive development of the frontal lobe adversely influences the everyday activities of children and adolescents, most of the previous research was conducted in clinical populations (e.g., ADHD children, schizophrenia patients) or focused on certain age ranges (e.g., 2–5 years of age) or developmental periods (e.g., early childhood) (16, 33, 34). Additionally, there is not enough converging evidence on the relationship between the structural maturation of the frontal lobe and the different domains of EFs during the transition between late childhood and adolescence. This may be mainly due to inconsistency in using neuroimaging techniques (e.g., electroencephalogram) and EF measures. For instance, a wide variety of intelligence quotient (IQ) tests in use today measure inconsistent domains of EFs (35, 36). That said, the Wechsler Adult Intelligence Scale III (WAIS-III), one of the most widely used IQ tests, measures a certain set of four mental abilities such as verbal comprehension, processing speed, perceptual organization, and working memory (35, 37). Considering this, an investigation of EFs by using multiple measures may be more beneficial to elucidate the relationship between the structural maturation of the frontal lobe and the development of the main domains of EFs in this population.

Considering the above concerns, we assessed regional GMVs (rGMVs) in the frontal lobe and its relationship with the main domains of EFs in a community sample of children and adolescents aged between 6 and 17 years. We used the voxel-based morphometry (VBM)–DARTEL procedure to measure GMVs in the frontal sub-regions and several EF tests to measure the different domains of EF. To assess the relationship, we conducted partial correlation analyses with four different age and sex groups (i.e., age ≤ 13 years, male; age ≤ 13 years, female; age ≥14 years, male; age ≥14 years, female) and performed multiple comparisons with these groups.

## Materials and Methods

### Participants

For participant recruitment, we used flyers that had brief information about the current study in schools and libraries in Seoul and Gyeonggi-do Province. We also recruited our participants from one elementary school and one high school in Seoul, South Korea. For the current study, we excluded participants if they had unstable physical condition or a history of an acquired brain injury, neurological disorders, psychiatric disorders (e.g., schizophrenia), developmental disorders (e.g., autism, intellectual disabilities), learning disabilities, language impairments, or uncorrected sensory impairment. All participants and their legal guardians gave their informed consent before taking part in the present study. Our study was approved by the Institutional Review Board for Human Subjects at Seoul National University Hospital and conducted according to the Declaration of Helsinki.

### Materials

#### Korean Educational Developmental Institute–Wechsler Intelligence Scale for Children

The Korean Educational Developmental Institute–Wechsler Intelligence Scale for Children (WISC) was a modified and standardized version of the original WISC for Korean children between 5 and 15 years of age (38–40). This test includes Full-Scale Intelligence Quotient (FSIQ) (i.e., a total score of the sub-tests), verbal IQ, and performance IQ, with 12 sub-tests: information, similarities, arithmetic, vocabulary, comprehension, picture completion, picture arrangement, block design, object assembly, and digital span and mazes (39). For our study, we used FSIQ to measure mental abilities of our participants who were 13 years or younger.

#### Korean-Wechsler Adult Intelligence Scale

We used the Korean version of the Wechsler Adult Intelligence Scale (K-WAIS), which is the most widely used IQ test with strong validity and reliability (41, 42). The K-WAIS has been widely used for Korean adolescents and adults whose ages are between 16 and 64 years. This test has two categories of 11 sub-tests with a verbal scale and a performance scale. The verbal scale includes information, digit span, vocabulary, arithmetic, comprehension, and similarity (42). The performance scale includes picture completion, picture arrangement, block design, object assembly, and digit symbol (42). For the current study, we used FSIQ to measure the mental abilities of our participants who were 14 years or older.

#### Stroop Color and Word Test

The Stroop test is one of the most widely used neurological tests to measure selective attention capacity and skills, cognitive flexibility, processing speed, and working memory (43–45). Moreover, it has been used to assess dysfunctions of the frontal lobe, as it has good validity and reliability in the pediatric and adolescent populations (44, 46). This test consists of three components such as word task, color task, and color–word task (46). The word task measures basic reading rate and assesses speech motor problems or learning disabilities. The color task assesses speech motor function and colorblindness. The color–word task measures both mental flexibility and inhibitory control. For the word task, participants were asked to name a series of color words (46). For the color task, participants were asked to name the color of cards (e.g., XXX in red or blue ink). For the color–word task, participants were asked to name the color of the ink instead of the word when the names of colors in different ink colors (e.g., the word “red” in blue ink) were shown. Three scores (i.e., word score, color score, color–word score) were calculated based on the number of completed items on each card. Higher scores indicate better performance and less interference. Interference score was also obtained by subtracting the obtained word score from the obtained color–word score (47). This score measures inhibitory control, with smaller scores indicating better control (47).

#### Advanced Test of Attention

Continuous performance test measures auditory attention, visual processing speed, visual-motor competence, and phonological awareness (48). It has good validity and reliability, with a Cronbach α coefficient of 0.82 (48–50). Among different versions of this test, we used the Advanced Test of Attention (ATA) that integrates both visual and auditory sensorial modalities, measuring attention, and response inhibition in Korean children older than 5 years (51). The target rate of stimulus presentation is 22% for the first section, 50% for the middle section, and 78% for the last section, with the stimulus presentation time of 0.1 s and an interval of 2 s between the presentations (39, 52). This test includes both auditory and visual scores of omission errors (i.e., missed targets), commission errors (i.e., incorrect responses to non-targets), the mean of response time (i.e., response speed), response time variability, response criterion, and detectability (i.e., ability to distinguish targets from non-targets) (39). It also provides both auditory and visual scores of ADHD. An omission error reflects sustained attention. A commission error reflects impulsivity, self-regulation, and inhibitory control. The mean of response time reflects the response preparation components of EF. Response time variability reflects the inconsistency in responses. Based on the ATA guidelines, higher scores on both the visual and auditory variables indicate higher probability of ADHD including poor EFs.

#### The Children's Depression Inventory

The Children's Depression Inventory (CDI), a modified version of Beck's Depression Inventory, measures child and adolescent depression (53–55). For our study, we used the Korean version (56) of the CDI, a self-report questionnaire with 27 items that reflect depressive symptoms, with numerical values from 0 to 2. Participants were asked to mark the statement that best described their feelings during the past 2 weeks (57). Higher scores reflect a higher severity of depression.

### Methods

#### MRI Data Acquisition

We used a 3.0-T MRI scanner (Siemens, Magnetom Tim-Trio) to obtain MRI scans. We obtained high-resolution T1-weighted images from each participant with a magnetization-prepared rapid acquisition gradient echo pulse sequence [repetition time = 1,900 ms, echo time = 3.13 ms, flip angle = 9°, matrix size = 256 × 256, field of view = 230 × 230 mm^2^, thickness = 0.9 mm]. We used foam pads to minimize head motion-related artifacts during scanning.

#### Regional VBM Analysis

To estimate rGMV, we conducted a VBM analysis through the SPM12 VBM-DARTEL procedure (SPM12, http://www.fil.ion.ucl.ac.uk/spm/, Wellcome Trust Center for Neuroimaging, London, UK) (58). Comparing with earlier optimized VBM, this procedure serves clearer segmentation and better registration for estimating boundaries between different tissues (58, 59).

Our well-trained physician did not find any abnormalities from motion and/or other artifacts on T1-weighted images. The procedure for preprocessing T1-weighted images included manual reorientation to the anterior commissure, gray matter segmentation based on a standard tissue probability map provided from SPM, creation of the study-specific template, spatial normalization with DARTEL to normalize individual images to the DARTEL template (34, 60, 61), modulation to adjust for volume signal changes during spatial normalization, and spatial smoothing of the gray matter partitions with a Gaussian kernel of 8-mm full width at half maximum. After preprocessing, values of rGMVs were extracted by averaging the values at each frontal region, according to the automated anatomical labeling atlas (62). The frontal sub-regions that we observed were the medial/inferior/superior parts of the orbitofrontal gyrus, the medial/middle/dorsal/superior parts of the frontal gyrus, opercular and triangular parts of the inferior frontal gyrus, and precentral gyrus in each hemisphere.

To conduct our study, we adapted a standard adult SPM template (62). Many neuroimaging studies on the developing brain have used the adult template and demonstrated that using the adult template did not affect the results of their analyses (63–67). Moreover, the standard adult SPM template is regarded as a good tool to compare or combine the results from previous studies across different age groups (68–70).

#### Statistical Analysis

Prior to analysis, we divided our sample into four age and sex groups (i.e., age ≤ 13 years, male; age ≤ 13 years, female; age ≥14 years, male; age ≥14 years, female), according to the guidelines of American Academy Pediatrics (71). We then proceeded to conduct partial correlation analyses between rGMVs in the frontal lobe and EF test scores (i.e., Stroop test, visual and auditory ATA) for each group. For the analyses, we controlled for age, sex, FSIQ, and TIV as covariates. We also adjusted for CDI in our final analyses.

Correlation values for each group were transformed into normal distributed values (i.e., *Z* group 1 = 0.5 × [log (1 + *R* group 1) – log (1 – *R* group 1)] and *Z* group 2 = 0.5 × [log (1 + *R* group 2) – log (1 – *R* group 2)] as Fisher *r*-to-*z* transformation). After *z* transformation, we compared them with *Z* = (*Z* group 1 – *Z* group 2) / √[1/(N group 1 – 3-*M*) + 1/(N group 2 – 3-*M*)], where N group 1, N group 2, and M each represent the sample size for each group and the number of covariates used in partial correlation analyses. A threshold of false discovery rate (FDR) = 0.2 was used to determine significant correlations and address multiple comparison issues (i.e., FDR = 0.2 or less) (72). FDR thresholding controls the expected proportion of false positives among brain areas exhibiting significance (72). FDR control levels in the range 0.1–0.2 are originally and practically known to be acceptable, as multiple neuroimaging studies have applied (72–76). We conducted all statistical analyses using the MATLAB-based custom software (MathWorks, Sherborn, MA, USA) and SPSS 20.0 for Windows (SPSS Inc., Chicago, IL, USA).

## Results

### Demographic and Clinical Characteristics of Participants

Overall, 123 participants aged between 6 and 17 years took part in our study. In our sample, the proportion of males (54.5%) was slightly larger than that of females (45.5%). The proportion of participants (56.9%) whose age was 13 years or younger was slightly larger than that of participants (43.1%) whose age was 14 years or older. The proportions of male and female participants in each year of age were also presented in Table 1. There were no sex differences on IQ and CDI in both age groups. However, sex differences on TIV were observed, as males had relatively larger TIV than females (Table 1). Table 2 shows executive functioning test performances in the four age and sex groups.

**Table 1:** Demographic and clinical characteristics of the participants.

**Characteristics**	**Total (*n* = 123)**	**Age** **≤13 years**			***p*^b^**	**Age** **≥14 years**			***p*^b^**
		**Total^a^ (*n* = 70)**	**Male (*n* = 35)**	**Female (*n* = 35)**		**Total^c^ (*n* = 53)**	**Male (*n* = 32)**	**Female (*n* = 21)**	
FSIQ^d^	102.23 (16.36)	104.03 (17.40)	100.57 (16.90)	107.49 (17.44)	0.097	99.65 (14.54)	97.13 (15.22)	104.00 (12.49)	0.095
TIV	1.42 (0.12)	1.43 (0.12)	1.47 (0.12)	1.40 (0.11)	0.007	1.41 (0.13)	1.45 (0.13)	1.36 (0.10)	0.006
CDI	14.81 (8.06)	10.72 (6.10)	11.53 (6.55)	9.97 (5.64)	0.302	20.08 (7.20)	19.19 (7.97)	21.50 (5.67)	0.228

*Values are means ± SD. Total n = 123; male n = 67 (54.5%); female n = 56 (45.5%); age ≤ 13 years, n = 7 0 (56.9%); age ≥14 years, n = 53 (43.1%)*.

a * Age ≤ 13 years group includes 6 years [n = 2; 2 males (100.0%), 0 females (0.0%)], 7 years [n = 6; 4 males (66.7%), 2 females (33.3%)], 8 years [n = 8; 2 males (25.0%), 6 females (75.0%)], 9 years [n = 10; 6 males (60.0%), 4 females (40.0%)], 10 years [n = 11; 7 males (63.6%), 4 females (36.4%)], 11 years [n = 11; 4 males (36.4%), 7 females (63.6%)], 12 years [n = 4; 1 male (25%), 3 females (75.0%)], and 13 years [n = 18; 9 males (50.0%), 9 females (50.0%)]*.

b * Two-tailed*.

c * Age ≥14 years group includes 14 years [n = 17; 9 males (52.9%), 8 females (47.1%)], 15 years [n = 14; 4 males (28.6%), 10 females (71.4%)], 16 years [n = 17; 14 males (82.4%), 3 females (17.6%)], and 17 years [n = 5; 5 males (100.0%), 0 females (0.0%)]*.

d * Korean Educational Developmental Institute–Wechsler Intelligence Scale for Children was used in age ≤ 13 years group and Korean-Wechsler Adult Intelligence Scale was used in age ≥14 years group. CDI, Children's Depression Inventory; FSIQ, Full-Scale Intelligence Quotient; TIV, total intracranial volume*.

**Table 2:** Executive functioning test performances across age and sex groups.

**Measures of executive function**	**Total (*n* = 123)**	**Age** **≤13 years**			**Age** **≥14 years**		
		**Total (*n* = 70)**	**Male (*n* = 35)**	**Female (*n* = 35)**	**Total (*n* = 53)**	**Male (*n* = 32)**	**Female (*n* = 21)**
ST word task	51.67 ± 20.14	50.09 ± 21.68	49.20 ± 21.49	50.97 ± 22.15	53.75 ± 17.90	54.53 ± 19.32	52.57 ± 15.87
ST color task	51.97 ± 16.46	54.19 ± 18.02	52.03 ± 15.62	56.34 ± 20.13	48.80 ± 13.50	48.68 ± 14.31	49.00 ± 12.39
ST color and word task	51.89 ± 17.78	55.79 ± 20.79	52.83 ± 21.85	58.74 ± 19.54	46.33 ± 10.10	47.00 ± 8.94	45.17 ± 12.03
ST interference	58.15 ± 15.57	58.73 ± 16.11	55.69 ± 17.50	61.77 ± 14.20	57.33 ± 14.89	59.23 ± 15.82	54.06 ± 12.89
aATA							
Omission errors	58.83 ± 19.38	66.34 ± 20.96	73.03 ± 21.48	59.65 ± 18.40	49.21 ± 11.49	49.94 ± 13.97	48.10 ± 6.21
Commission errors	64.60 ± 19.14	68.94 ± 21.09	74.12 ± 21.95	63.76 ± 19.12	59.02 ± 14.69	55.94 ± 12.01	63.71 ± 17.29
Mean of response time	60.24 ± 14.19	57.71 ± 14.04	60.15 ± 11.86	55.26 ± 15.73	63.49 ± 13.84	63.41 ± 15.76	63.62 ± 10.63
Response time variability	56.04 ± 19.84	61.16 ± 20.00	68.53 ± 21.86	53.79 ± 14.92	49.47 ± 17.73	46.78 ± 14.51	53.57 ± 21.49
ADHD score	120.39 ± 22.46	126.57 ± 24.40	134.85 ± 26.42	118.29 ± 19.21	112.45 ± 16.84	110.88 ± 14.07	114.86 ± 20.50
Detectability	3.26 ± 1.16	2.91 ± 1.15	2.64 ± 1.13	3.17 ± 1.12	3.70 ± 1.01	3.71 ± 1.10	3.68 ± 0.87
Response criterion	0.78 ± 0.85	0.83 ± 0.70	0.93 ± 0.71	0.73 ± 0.69	0.72 ± 1.01	0.81 ± 1.22	0.58 ± 0.56
vATA							
Omission errors	71.17 ± 22.13	72.91 ± 20.59	75.44 ± 19.12	70.38 ± 21.96	68.94 ± 23.97	66.25 ± 24.92	73.05 ± 22.40
Commission errors	71.71 ± 21.02	75.60 ± 19.88	78.32 ± 18.82	72.88 ± 20.81	66.72 ± 21.58	63.38 ± 21.05	71.81 ± 21.88
Mean of response time	50.07 ± 15.37	44.07 ± 15.11	42.71 ± 16.40	45.44 ± 13.82	57.77 ± 11.98	56.41 ± 14.29	59.86 ± 7.01
Response time variability	49.56 ± 10.06	50.04 ± 10.38	51.12 ± 9.79	48.97 ± 10.97	48.94 ± 9.69	46.44 ± 9.82	52.76 ± 8.31
ADHD score	126.34 ± 23.35	128.31 ± 21.66	131.09 ± 20.43	125.53 ± 22.80	123.81 ± 25.35	120.56 ± 25.66	128.76 ± 24.64
Detectability	2.23 ± 1.33	1.69 ± 1.22	1.45 ± 1.12	1.93 ± 1.28	2.92 ± 1.13	3.13 ± 1.22	2.61 ± 0.92
Response criterion	0.91 ± 0.55	0.87 ± 0.38	0.87 ± 0.33	0.86 ± 0.42	0.96 ± 0.71	1.08 ± 0.83	0.78 ± 0.42

*Values are means ± SD. aATA, auditory ATA; ST, Stroop test; vATA, visual ATA*.

### Correlation Analysis

**Figure 1:**
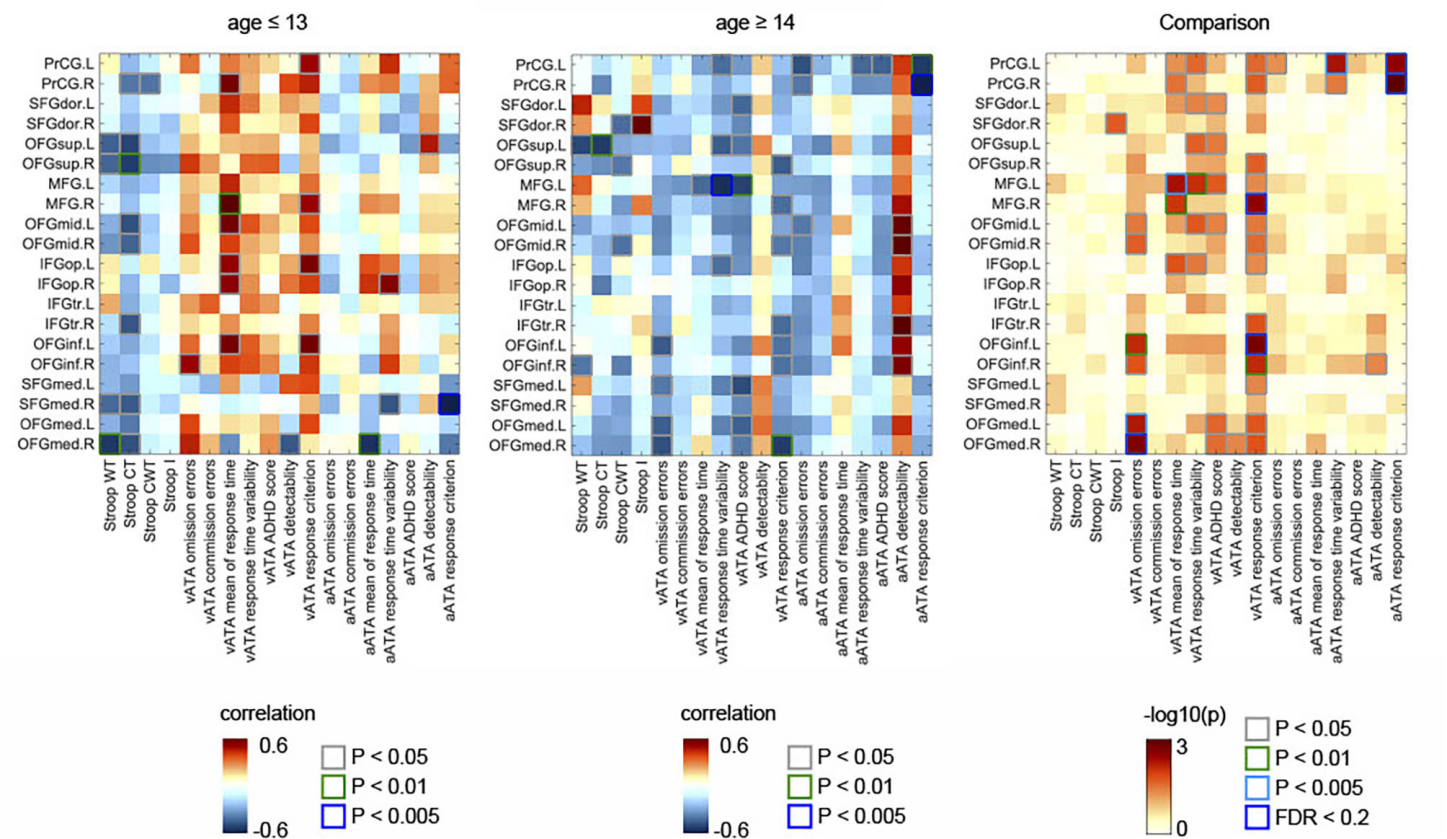
Partial correlations between the GMVs in the frontal lobe and Stroop and ATA test scores in the age groups (age ≤ 13 years, *n* = 70; age ≥14 years, *n* = 53). Comparisons of the partial correlations of the age groups. Controlled for sex, FSIQ, and TIV. GMVs, gray matter volumes; FSIQ, Full-Scale Intelligence Quotient; TIV, total intracranial volume; Stroop WT, Stroop Word Test; Stroop CT, Stroop Color Test; Stroop CWT, Stroop Color and Word Test; Stroop I, Stroop inference score; aATA, auditory ATA; vATA, visual ATA.

As shown in Figure 1, in the age group ≤ 13 years, we found numerous negative correlations and a few positive correlations between the GMVs in the frontal sub-regions and Stroop test and ATA scores (controlled for sex, FSIQ, and TIV). Contrastingly, in the age group ≥14 years, we found many positive correlations between the GMVs and ATA scores, with a few negative correlations of the GMVs with Stroop test scores (Figure 1). Even after FDR correction (i.e., FDR <0.2), we found multiple significant correlations in each age group. However, the FDR-corrected correlations did not withstand adjustment for CDI.

**Figure 2:**
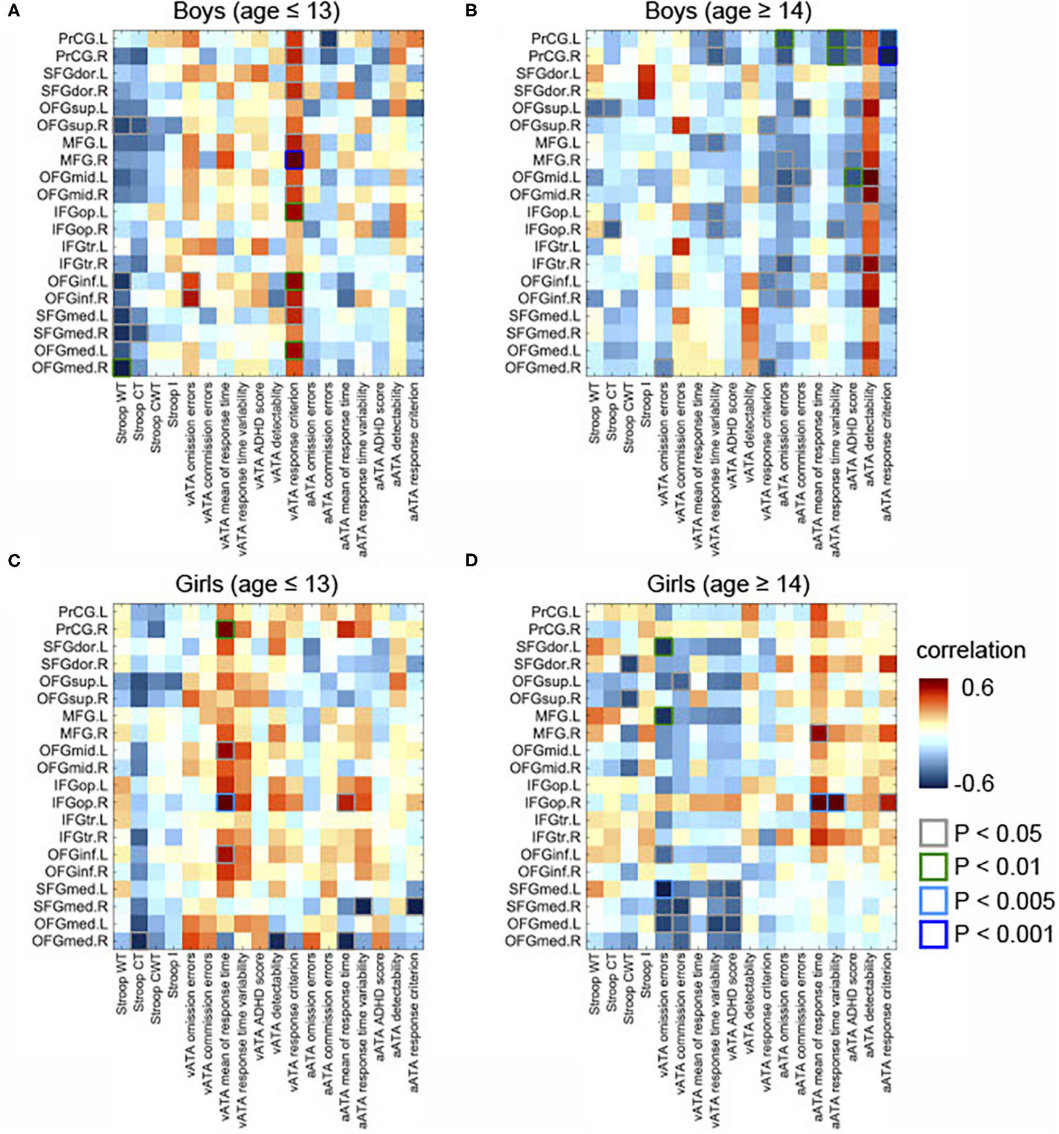
Partial correlations between the GMVs in the frontal lobe and Stroop and ATA test scores in the age and sex groups. Controlled for age, FSIQ, and TIV. **(A)** Male group (age ≤ 13 years, *n* = 34). **(B)** Male group (age ≥14 years, *n* = 31). **(C)** Female group (age ≤ 13 years, *n* = 34). **(D)** Female group (age ≥14 years, *n* = 18). GMVs, gray matter volumes; FSIQ, Full-Scale Intelligence Quotient; TIV, total intracranial volume; Stroop WT, Stroop Word Test; Stroop CT, Stroop Color Test; Stroop CWT, Stroop Color and Word Test; Stroop I, Stroop inference score; aATA, auditory ATA; vATA, visual ATA.

**Figure 3:**
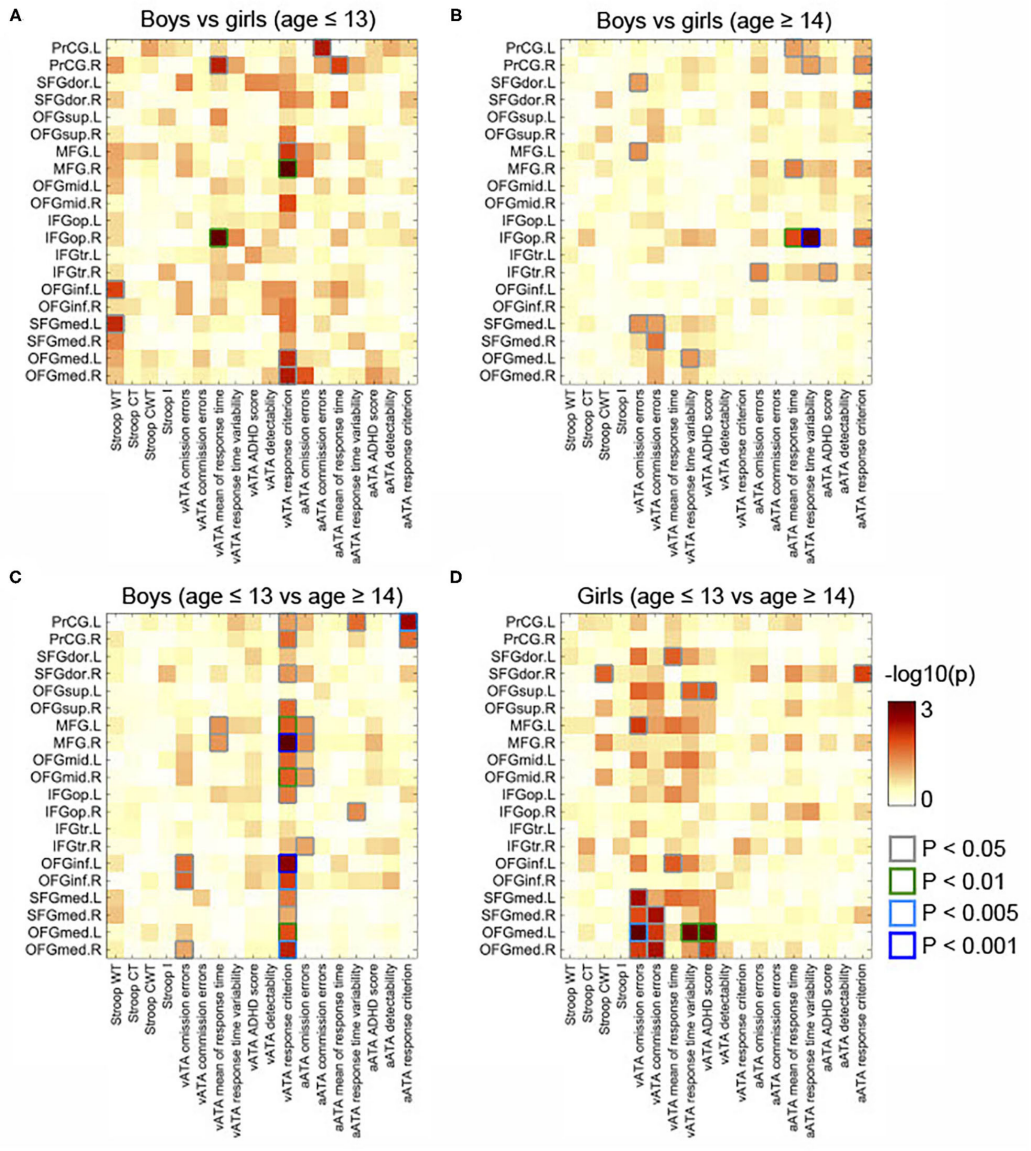
Comparisons of partial correlations between the GMVs in the frontal lobe and Stroop and ATA test scores in the age and sex groups. Controlled for age, FSIQ, and TIV. **(A)** Male group vs. female group (age ≤ 13 years). **(B)** Male group vs. female group (age ≥14 years). **(C)** Male group (age ≤ 13 years) vs. male group (age ≥14 years). **(D)** Female group (age ≤ 13 years) vs. female group (age ≥14 years). GMVs, gray matter volumes; FSIQ, Full-Scale Intelligence Quotient; TIV, total intracranial volume. Stroop WT, Stroop Word Test; Stroop CT, Stroop Color Test; Stroop CWT, Stroop Color and Word Test; Stroop I, Stroop inference score; aATA, auditory ATA; vATA, visual ATA.

As displayed in Figure 3C, we found significant differences between the results age ≤ 13 group and those of age ≥14 male groups (controlled for sex, FSIQ, TIV). Specifically, the GMVs in many frontal sub-regions were negatively correlated with ATA scores in the age group ≤ 13 years (Figure 2A) but were positively correlated with ATA scores, mainly with visual scores, in the age group ≥14 years (Figure 2B). Among these significant correlations, the correlations that survived for FDR correction were the left precentral gyrus and auditory ATA response criterion, the right middle frontal gyrus and visual ATA response criterion, and the left inferior orbitofrontal gyrus and visual ATA response criterion (FDR = 0.1535, 0.0867, and 0.1351, respectively). However, after adjustment for CDI, only the correlation between the right middle frontal gyrus and visual ATA response criterion survived FDR correction (FDR = 0.1857), as shown in **Figure 5C**. Similarly, we found significant differences between the age ≤ 13 female group and age ≥14 female group (Figure 3D). Even after controlling for CDI, we found multiple significant correlations of the rGMVs with ATA scores, although the magnitude of these correlations did not withstand FDR correction (**Figure 5D**).

#### Age Group ≤ 13 Years

**Figure 4:**
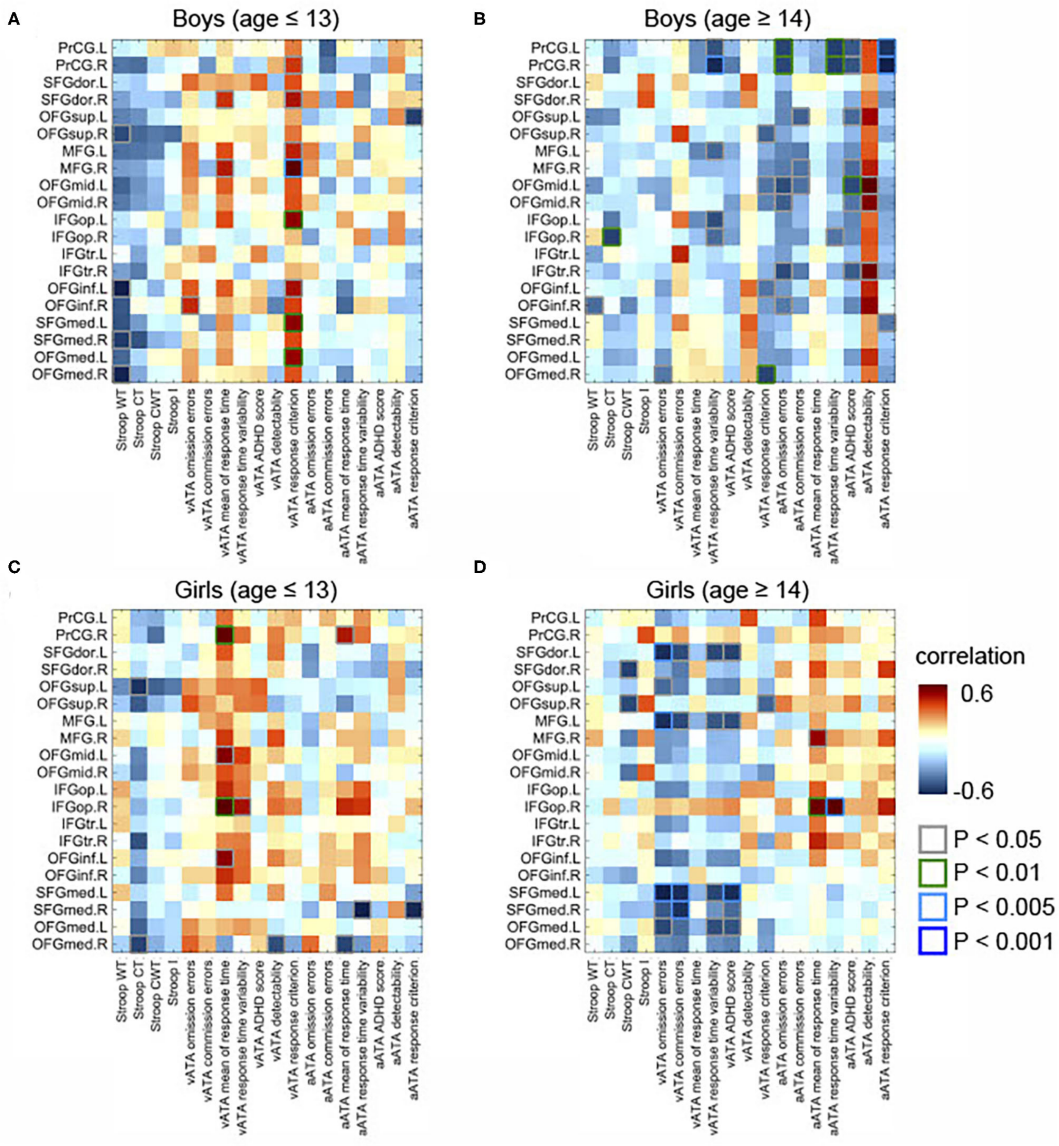
Partial correlations between the GMVs in the frontal lobe and Stroop and ATA test scores in the age and sex groups. Controlled for age, FSIQ, TIV, and CDI. **(A)** Male group (age ≤ 13 years, *n* = 34). **(B)** Male group (age ≥14 years, *n* = 31). **(C)** Female group (age ≤ 13 years, *n* = 34). **(D)** Female group (age ≥14 years, *n* = 18). GMVs, gray matter volumes; FSIQ, Full-Scale Intelligence Quotient; TIV, total intracranial volume; Stroop WT, Stroop Word Test; Stroop CT, Stroop Color Test; Stroop CWT, Stroop Color and Word Test; Stroop I, Stroop inference score; aATA, auditory ATA; vATA, visual ATA.

In male participants, we found that rGMVs in many frontal sub-regions were negatively associated with Stroop test and ATA scores (controlled for age, FSIQ, and TIV) (Figure 2A). The negative correlations that had significant evidence (i.e., *p* < 0.01 or *p* < 0.005 or *p* < 0.001) were observed in the following: the right middle frontal gyrus and visual ATA response criterion (*p* < 0.001), the left opercular inferior frontal gyrus and visual ATA response criterion (*p* < 0.001), the left inferior orbitofrontal gyrus and visual ATA response criterion (*p* < 0.01), the left medial orbitofrontal gyrus and visual ATA response criterion (*p* < 0.01), and the right medial orbitofrontal gyrus and Stroop Word Test score (*p* < 0.01). However, as displayed in Figure 4A, adjustment for CDI decreased the magnitude of the following negative correlations: the right middle frontal gyrus and visual ATA response criterion (*p* < 0.005), the left inferior orbitofrontal gyrus and visual ATA response criterion (*p* < 0.05), and the right medial orbitofrontal gyrus and Stroop Word Test score (*p* < 0.05). Adjustment for CDI increased the magnitude of the correlation between the left medial superior frontal gyrus (left) and visual ATA response criterion (*p* < 0.01). The strength of the correlations between the left opercular inferior frontal gyrus and visual ATA response criterion and the left medial orbitofrontal gyrus and visual ATA response criterion remained the same (*p* < 0.01). However, such results did not withstand FDR correction.

In female participants, there were several significant correlations (adjusted for age, FSIQ, and TIV), as shown in Figure 2C. Among the correlations that had strong evidence, visual ATA mean of response time was strongly correlated with the right precentral gyrus and the right opercular inferior frontal gyrus (*p* < 0.01 and *p* < 0.005, respectively). After adjustment for CDI, the strength of the correlation between the right precentral gyrus and visual ATA mean of response time remained the same, whereas the strength of the correlation between the right opercular inferior frontal gyrus and visual ATA mean of response time was decreased slightly (*p* < 0.01) (Figure 4C). However, such results did not survive FDR correction.

**Figure 5:**
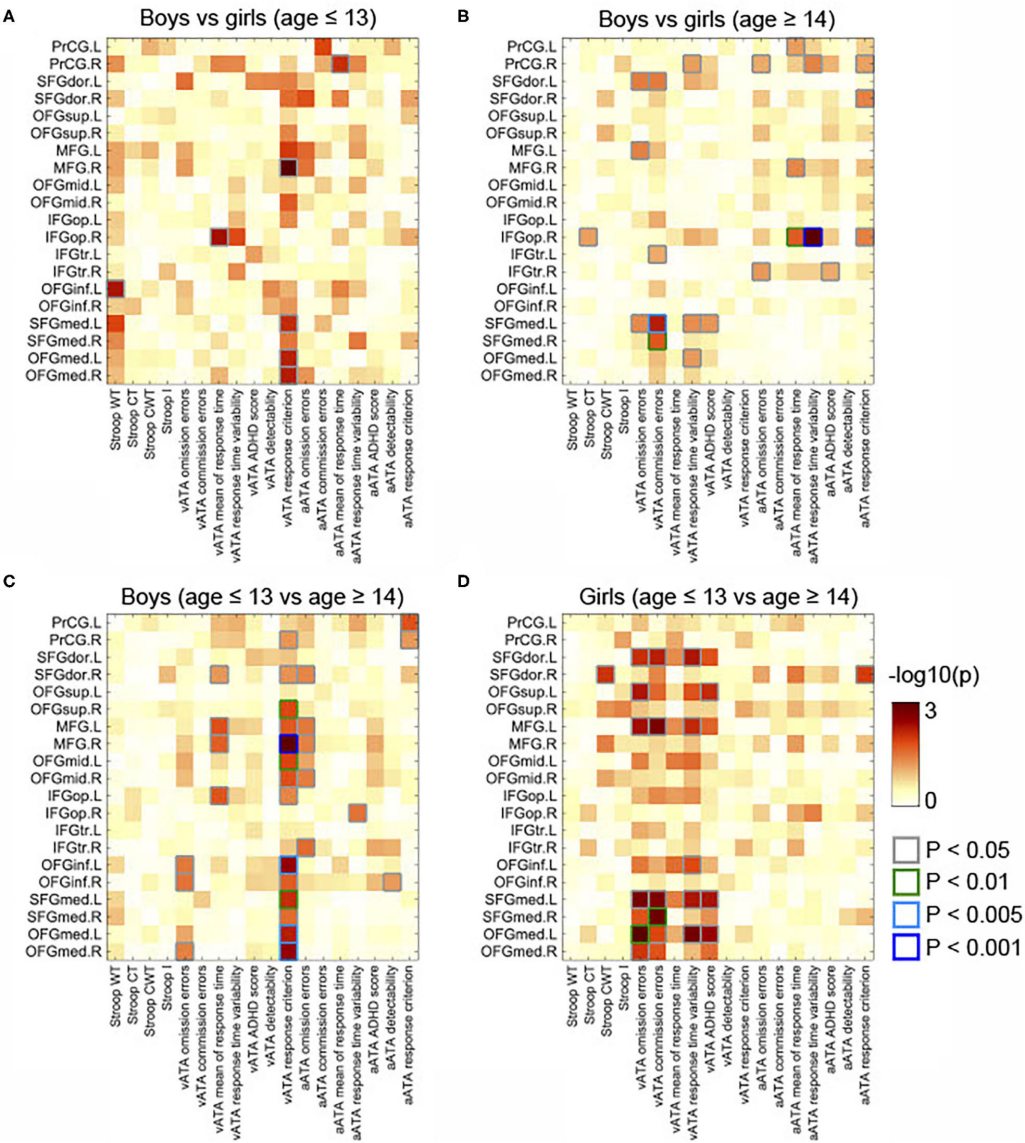
Comparisons of partial correlations between the GMVs in the frontal lobe and Stroop and ATA test scores in the age and sex groups. Controlled for age, FSIQ, TIV, and CDI. **(A)** Male group vs. female group (age ≤ 13 years). **(B)** Male group vs. female group (age ≥14 years). **(C)** Male group (age ≤ 13 years) vs. male group (age ≥14 years). **(D)** Female group (age ≤ 13 years) vs. female group (age ≥14 years). GMVs, gray matter volumes; FSIQ, Full-Scale Intelligence Quotient; TIV, total intracranial volume; Stroop WT, Stroop Word Test; Stroop CT, Stroop Color Test; Stroop CWT, Stroop Color and Word Test; Stroop I, Stroop inference score; aATA, auditory ATA; vATA, visual ATA.

Additionally, we found sex differences on multiple correlations in this age group (Figure 3A). Most of these correlations were strongly associated with both visual and auditory ATA scores, although the adjustment for CDI slightly reduced the magnitude of the sex differences (Figure 5A). Both the results with CDI and without CDI did not withstand FDR correction.

#### Age Group ≥14 Years

After adjustment for age, FSIQ, and TIV, we found strong evidence on many positive correlations between the GMVs and ATA scores, mainly with auditory ATA scores, in male participants (Figure 2B). Among the correlations, the correlations that had significant evidence (i.e., *p* < 0.01, *p* < 0.005, or *p* < 0.001) were identified in the following: the left precentral gyrus and visual ATA detectability (*p* < 0.01), the left precentral gyrus and auditory ATA mean of response time (*p* < 0.01), the left precentral gyrus and auditory ATA response criterion (*p* > 0.005), the right precentral gyrus and auditory ATA mean of response time (*p* < 0.01), the right precentral gyrus and auditory ATA response criterion (*p* < 0.001), and the left middle orbitofrontal gyrus and auditory ATA score (*p* < 0.01). After adjusting for CDI, all the correlations remained significant, and the positive correlation between the right medial orbitofrontal gyrus and visual ATA response criterion became significant (Figure 4B). There were no significant correlations of the rGMVs with Stroop test scores.

Similarly, we found that the rGMVs in some frontal sub-regions were positively associated with visual ATA scores; Stroop test and auditory ATA variables were not strongly correlated with the rGMVs (Figure 2D). The correlations that had significant evidence include the left dorsolateral superior frontal gyrus and ATA omission errors (*p* < 0.01), the left middle frontal gyrus and ATA omission errors (*p* < 0.01), the right opercular inferior frontal gyrus and ATA mean of response time (*p* < 0.005), the right opercular inferior frontal gyrus and ATA response time variability (*p* < 0.005), and the left medial superior frontal gyrus and ATA omission errors (*p* < 0.005). Even after adjusting for CDI, most of the correlations remained strong and significant (Figure 4D).

The correlations that showed sex differences in this age group are shown in Figure 3B. Specifically, among those, the correlation between the right opercular inferior frontal gyrus and auditory ATA mean of response time and the correlation between right opercular inferior frontal gyrus and auditory ATA response time variability had significant evidence (*p* < 0.01 and *p* < 0.001, respectively). Only the correlation between the right opercular inferior frontal gyrus and auditory ATA response time variability survived FDR correction (FDR = 0.0992). The sex difference on this correlation remained strong even after we controlled for CDI and performed FDR correction (FDR = 0.0912) (Figure 5B). After adjusting for CDI, the magnitude of sex differences was increased in the correlation between the left medial superior frontal gyrus and visual ATA commission errors (*p* < 0.005) and the correlation between the right medial superior frontal gyrus and visual ATA commission errors (*p* < 0.01) (Figure 5B).

## Discussion

At present, although the development of the frontal lobe has been extensively investigated, it is still unclear about how the structural development of the frontal lobe is associated with different executive functioning skills in late childhood and adolescence. To fill the gap in evidence in this specific area, we examined the relationship between GMVs in the frontal sub-regions and the main domains of EFs in late childhood and adolescence. Interestingly, we found that most of the visual and auditory ATA scores that mainly measured attentional control were negatively correlated with the rGMVs in participants in late childhood and positively correlated with the rGMVs in participants in adolescence. Only a few correlations of the GMVs with Stroop test scores that assessed various higher-order EFs were strong and significant in both age groups (i.e., age ≤ 13 years, age ≥14 years). Even after adjusting for CDI, we observed the similar results.

Moreover, in both age groups, sex differences were shown in the majority of the correlations with ATA scores, whereas only a few correlations with Stroop test scores showed sex differences. The only correlation that survived FDR correction even after controlling for CDI was the right opercular inferior frontal gyrus and auditory ATA response time variability [FDR = 0.0992 (without CDI), FDR = 0.0912 (with CDI)] in participants in adolescence. Based on such findings, we suggest that the sex differences might reflect the earlier structural maturation of the frontal lobe in females, and specifically, attentional control, which is one of the main forms of EFs, might develop earlier in females. Strictly, although most of our findings were no longer significant after FDR correction (i.e., FDR <0.2), considering the potentially significant novelty of our study, here we present and discuss our findings based on uncorrected *p*-values (i.e., *p* ≤ 0.05).

The results of our correlation analyses showed a statistically significant developmental trend in several domains of attention (i.e., selective attention, sustained attention), as there were numerous negative correlations of the rGMVs in the frontal sub-regions with ATA test performances in participants in late childhood in the current study. We speculate that this might possibly reflect ongoing significant maturation of the frontal sub-regions involved in attention, as GMV reduction is one of the major characteristics of adolescent brain maturation (77). On the other hand, a few Stroop test scores that assessed the domains of higher-order EFs (e.g., working memory) were strongly correlated with the GMVs in the frontal sub-regions, which may possibly present the possibility of the later maturation of higher-order executive functioning skills.

The important roles of attention include alertness, set, spatial attention, sustained attention, and interference control (9). These roles seem to develop gradually toward full maturity at 12 years of age (9, 78). In line with this, our results revealed the peak of the development of these domains in late childhood, and presented the possibility that the development of attention might already have passed the peak and become stable during this developmental period. Furthermore, attention is known to have distinctive functions (78). Posner and Rothbart (79) proposed a model of attentional networks. In this model, there are three distinctive networks such as the alerting network, the orienting network, and the executive attention network (79–81). In line with this model, all frontal sub-regions had strong evidence with the executive skills of visual and auditory attention in our participants. Considering this, we suggest that the GMVs in the frontal sub-regions that are implicated with the attentional networks may dramatically develop during late childhood.

Among the frontal sub-regions we assessed, the superior frontal gyrus, the orbitofrontal gyrus, the middle frontal gyrus, and the inferior frontal gyrus are involved in the executive attention network. The executive attention network is primarily responsible for error detection and the goal-directed control of attention and behavior associated with novelty and interference (81, 82). This network is active when a conflict is present and inhibitory attentional processes are necessary (81, 83). It is known that while the development of executive attention (e.g., alertness from external cues) occurs since early infancy, the development of orienting, and maintaining the level of alertness seems to develop through late childhood (78). This was well-demonstrated in our study, as our findings revealed the structurally developmental changes in the frontal sub-regions and the growth spurt in the domains of attentional control in participants in late childhood.

We also observed the structurally significant development of the alerting network in the GMV in the precentral gyrus, which has a main role in executing voluntary movements (78). The alerting network is mainly involved in achieving and maintaining a state of high sensitivity to external stimuli (78). This network is considered as one of the prerequisites of conscious perception and other attentional operations (78, 83). The development of this network continues beyond 14 years of age (83). Our findings are consistent with this notion, as there was a large number of positive correlations between the GMV in the precentral gyrus and both visual and auditory attentional task performances in our participants who were 14 years or older. More importantly, there was also statistically strong evidence (FDR = 0.01535; controlled for sex, TIV, and FSIQ) on the correlation between the left precentral gyrus and auditory attentional response criterion when we compared the result of male participants in late childhood with that of male participants in adolescence. That is, male participants in the age group ≥14 years whose GMV in the precentral gyrus was more developed responded less impulsively than male participants in age ≤ 13 male group while they performed the auditory attentional tasks.

Combined with previous neuroimaging evidence and the model of attentional networks, we suggest that GMVs in most of the frontal sub-regions that are associated with the attentional networks might significantly develop in late childhood and become stabilized in early adolescence. Furthermore, it is possible that the maturation of higher-order executive functioning skills might occur later in adolescence, as we did not find any strong evidence on the relationship between structural maturation of the frontal sub-regions and Stroop test task performances.

Despite the novelty of our findings, our study has several limitations that merit attention. First, our study was exploratory, and our findings were based on uncorrected *p*-values, as most of our findings did not survive FDR correction. However, this may possibly be due to the limitation of the relatively low sample size, which is one of the common limitations of neuroimaging studies. We therefore recommend future studies to obtain larger sample sizes with more balanced proportions of male and female participants to better understand how rGMVs in the frontal lobe contributes to a wide range of EFs in late childhood and adolescence. Moreover, measuring a single executive skill is difficult, as all aspects of EFs are inter-twined (84). It is also possible that other potential covariates (e.g., stress) might have contributed to the strength of the statistically significant correlations we found, although we controlled for the well-known covariates in our analyses (85, 86). Lastly, the nature of our study (i.e., the cross-sectional design) does not provide any further evidence on the causal relationship between the associations found. All cross-sectional studies may be subject to the bias of the selection of people of various ages. Considering this, it may be beneficial to longitudinally investigate how the structural maturation of the frontal lobe is linked to the different domains of executive functioning skills in late childhood and adolescence. Although our findings were based on uncorrected *p*-values, our findings may provide better insights into elucidating the structural maturation of the frontal lobe and its relationship with the development of attention networks in late childhood and adolescence.

## Data Availability Statement

The raw data supporting the conclusions of this article will be made available by the authors, without undue reservation.

## Ethics Statement

The studies involving human participants were reviewed and approved by The Institutional Review Board for Human Subjects at Seoul National University Hospital. Written informed consent to participate in this study was provided by the participants' legal guardian/next of kin.

## Author Contributions

M-HP designed the study and wrote the protocol. DS, BK, B-NK, SP, K-IJ, HK, and S-YL conducted the literature searches and analyses. M-HP, BP, and DS performed the statistical analysis. DS prepared Tables 1 **and** 2. BP prepared figures. DS and BP wrote the first and final draft of the manuscript. All authors have approved the final manuscript.

## Conflict of Interest

The authors declare that the research was conducted in the absence of any commercial or financial relationships that could be construed as a potential conflict of interest.
